# OCT on Anterior Segment Anomalies of the Eye in a Polish Paediatric Cohort: Diagnostic and Therapeutic Challenges

**DOI:** 10.1155/2021/6646098

**Published:** 2021-02-18

**Authors:** Katarzyna Lepska, Dariusz Dobrowolski, Katarzyna Krysik, Anita Lyssek-Boroń, Edward Wylęgała

**Affiliations:** ^1^Department of Ophthalmology, St. Barbara Hospital, Trauma Centre, Medykow Square 1, 41200 Sosnowiec, Poland; ^2^Chair and Clinical Department of Ophthalmology, Division of Medical Science in Zabrze, Medical University of Silesia, Panewnicka 65 Str., 40760 Katowice, Poland; ^3^Department of Ophthalmology, District Railway Hospital, Panewnicka 65 Str., 40-760 Katowice, Poland; ^4^Faculty of Medicine, University of Technology, Rolna 43 Str., 40-555 Katowice, Poland

## Abstract

**Purpose:**

To present applications of anterior segment optical coherent tomography (AS OCT) for anomalies of the eye in a Polish paediatric cohort.

**Materials and Methods:**

Seventy-four eyes of infants and older children were examined. The majority of them underwent general anaesthesia to allow OCT to be performed in the operating room, but a few were examined in a routine way. We focused on corneal, anterior chamber, iris, and lens disorders. Measurements included corneal morphology, anatomy of the anterior chamber, and general involvement of surrounding tissues in pathologic lesions.

**Results:**

We divided the paediatric patients into several groups by considering the type of disease and involvement of particular tissues. The groups were selected based on OCT usefulness in describing their ocular disorders.

**Conclusion:**

The collected anterior segment disorders showed huge usefulness for paediatric diagnosis and treatment planning.

## 1. Introduction

Ocular anterior segment abnormalities are rare disorders involving various tissues. Isolated changes and disorders form complexes of various irregularities. Some of these originate from genetic-related diseases, but there are also disturbances revealed during organogenesis as a result of external factors. A separate group consists of injuries and inflammatory changes affecting the youngest patients.

In recent years, anterior segment optical coherent tomography (AS OCT) has become the basic diagnostic method for planning ambulatory and surgical treatment [1]. The development of medical devices has allowed the application of this diagnostic method in paediatric patients in the operating room during general anaesthesia, enabling the precise assessment and detailed correlation of visible changes during ophthalmoscopic examinations with OCT imaging. This type of examination is highly valuable, especially when planning surgical treatment [2].

Over the last 10 years, we have been developing a paediatric keratoplasty programme that is aimed at restoring or improving vision in eyes with corneal disorders or diseases involving the whole anterior segment of the eye. During candidate selection, we admitted children with various abnormalities for diagnosis, many of whom were suffering from diseases affecting the anterior segment of the eye with the involvement of various tissues and with various levels of severity.

In infants, the diagnostic process is complicated because of the limited number of devices that can be readily used with them. Recently, anterior segment imaging has been improved with the introduction of AS OCT, first fixed on special holders and now integrated into surgical microscopes. In older children, stationary devices can be used, but children's cooperation during the procedure can be problematic; therefore, general anaesthesia is useful in this group. Its main application is for the examination of children with Down syndrome who present psychosomatic disorders, as they often do not allow themselves to be subjected to any form of diagnostic imaging.

The study group on which OCT was performed can be divided depending on the nature of the changes involved. The fundamental problem is the transparency of the optical media. In OCT imaging, there is an issue of whether transparency loss concerns only the cornea or only the lens, whether other structures of the anterior segment are involved, or whether additional structures, such as congenital or acquired membranes in the anterior chamber, are involved. Even with partially transparent corneas, most pathologic changes can be observed during an ophthalmoscopic examination. OCT is applied to locate lesions precisely; if their location is inside corneal layers, the visualisation of additional disorders, such as anterior synechiae, may be difficult. OCT scans also help in locating the lens and finding its structural disorders. Infants and young patients with coexisting glaucoma may also experience benefits related to OCT imaging, which can not only support diagnostics and explain the cause of the disease but also monitor the effectiveness of the therapy [3]. Additionally, it plays a key role in the diagnosis of Peters anomaly. The examination allows for an accurate assessment of the anterior segment and is crucial for selecting the best treatment method.

## 2. Materials and Methods

Seventy-two children who had been referred for diagnosis and treatment because of corneal or anterior segment disorders were examined. OCT of the anterior segment under general anaesthesia was performed on children ranging in age from 2 months to 6 years; older children were commonly examined using stationary devices. Children with mental disabilities aged above 6 years were examined under anaesthesia. The lack of cooperation in older children meant that the examination was performed under anaesthesia. Examinations, if necessary, were repeated every 6 or 12 months.

The main indication was local or total reduced corneal transparency, but more severe cases included the involvement of the iris or the lens. Anterior segment anomalies were also observed, even if there were no corneal abnormalities. To establish the evolution of changes and treatment accuracy, postinflammatory leucomas or posttraumatic lesions were observed. We selected the main disorders for examination during the diagnostic process and combined them with the most common diseases. The selection was focused on the expected measurements that were necessary to support diagnosis. Some of these pointed out the structure of the corneal layers, others indicated the width and depth of particular lesions, and others showed shape factors, including the anterior curvature, the posterior curvature, the shape of the anterior chamber, and the positions of the iris and the lens. Each exam was unique because of the high diversity of corneal abnormalities and the combined lesions of tissues around the anterior chamber.

## 3. Results

Our observations show the significant diversity of changes observed in the anterior segment of the eye. The use of OCT in diagnosis significantly broadens our knowledge and allows for better treatment planning. Determining the location of lesions and the topography of the predisposition of the eye is the main advantage of imaging. The detailed pathologies of OCT imaging are presented in Table 1.

The biggest challenge in diagnosis and the planning of treatments is the eyes of newborns, which have nontransparent corneas. Before the use of OCT, we did not have an effective imaging method. Ultrasound imaging and UBM are commonly used methods; however, due to too their low resolution, they often cannot provide precise diagnostic answers. Therefore, in congenital defects in which we have a complete loss of corneal transparency, OCT allows us to trace changes in tissues.

At the corneal level, we can very accurately assess the entire structure of its stroma. This is extremely important due to the need to assess the thickness of the tissue and the width and depth of the opaque tissue area. In congenital or acquired loss of corneal transparency, OCT imaging shows the exact location of scars, fibrosis, or leucoma in particular layers of the cornea.

Another important aspect is the assessment of the corneal stroma's vascular network, which affects not only the superficial part of the cornea but also the anterior or deep stroma. It is well known that the presence of vascularized tissues makes the risk of failure of keratoplasty very high, especially in young children. The presence of neovascularization supports a high tendency to fibrosis where the pathological vessels are formed. An assessment of the degree of vascularization strongly interferes with planning surgical management.

The surface is usually well seen. The interface between the cornea and the sclera in congenital disorders presents pathological vascularization affecting the limbal epithelium. We can expect the features of the limbal insufficiency and irregular location of the conjunctival vessels on the surface of the cornea. The palisades of Vogt are not detectable, which is a serious obstacle for properly locating a keratoplasty.

Taken together, the condition of the corneal epithelium, the presence of tissues of conjunctival origin, the condition and structure of the corneal stroma, the vascularity of the corneal stroma and its shape, and corneal thickness are important prognostic factors in the planning of surgical interventions. Placing of corneal stromal thinning or irregular anterior and posterior curvature determines the area of planned surgical management. All these features can influence a planned surgery, while also implying certain consequences during and shortly after surgical treatment in the case of postinflammatory or acquired disorders. In posttraumatic eyes, we usually deal with a smaller range of changes than in the case of acquired diseases. The postinflammatory opacities themselves mainly affect the cornea, much less often are their changes that reach the tissues of the lens, iris, and iridocorneal angle.

Postinflammatory leucomas often develop vascularization of the ocular surface and can also present deep vascularization of the stroma. Vessels that run on the posterior corneal surface under the Descemet layer can also be found. The condition and range of this vascularity may be crucial for planning the procedure. If we find multifocal neovascularization, an active inflammatory process, and tissue hypoxia, surgical intervention may be possible after additional anti-inflammatory treatment. Early surgery, as a consequence of an intervention in the inflamed area, may lead to early rejection of the transplanted tissue.

Changes involving the iris and the lens can also be connected with the posterior corneal surface, e.g., in the case of Peters anomaly. We usually observe adhesions around the pupil, while in more advanced cases, the lens and iris form an entire complex with the cornea; the iris and lens are solidified with each other. Of course, the latter situation necessitates quick intervention, especially when it takes place bilaterally. Otherwise, if the disease affects only the cornea with accompanying mild abnormalities and the transparency of the tissue is satisfactory, conservative treatment may be considered. However, thanks to OCT examinations, we can very precisely assess the location of cornea oedema, adhesions, and irregularities on the surfaces of the cornea. The changes that touch the posterior curvature are less important for the development of vision in a child. With OCT, we can not only assess the shape of the cornea and extend an examination over its entire structure but also determine the defects occurring in the visual axis—the key to obtaining good visual acuity in young patients.

An OCT examination provides additional information on the filtration angle. In both congenital and acquired defects, there is a tendency for the formation of adhesions reaching the infiltration angle. Such changes favour the formation of glaucoma, especially in inflammatory diseases or posttraumatic ones.

A separate category is patients who have undergone previous surgeries for corneal transplants or cataract removal. They may experience secondary changes in the anterior segment: the formation of anterior adhesions with subsequent glaucoma, formation of retrocorneal or retropupil membranes, or the fibrosis of the lens capsule. The abovementioned changes are secondary; if they are accompanied by a reduction in transparency of the corneas, keratoplasty should be considered with anterior chamber plastic. Many of them are difficult to observe in the slit lamp; therefore, tomography brings additional advantages that allow tracking not only the actual state but also its dynamics. For some diseases, especially congenital, the condition of the cornea evolves with the growth of the globe; therefore, long-term observation can provide additional information and prevent either premature or undue interventions.

**Figure 1:**
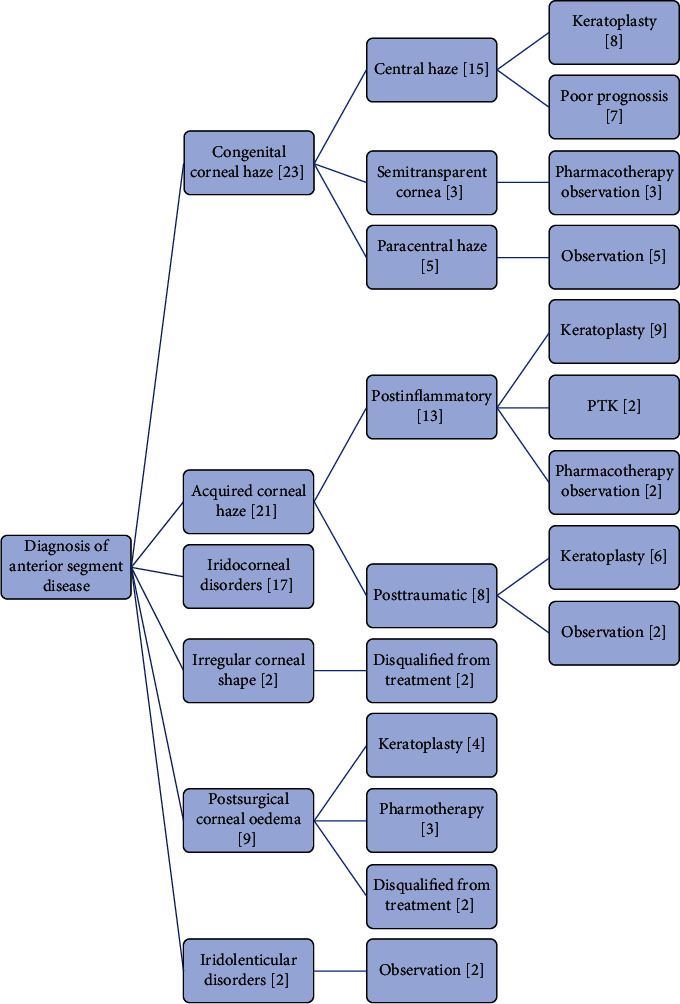
Scheme of the procedure and qualification for further treatment of the studied patients.

Our observations are presented in the accompanying graphic (Figure 1). We try to refer to the method of deciding on the procedure and the conditions indicated by the OCT examination. This scheme allows us to understand how we examine patients and why they do or do not qualify for surgery. It also acts as a guide on assessing the risk of intervention based on an OCT examination. In the presented diagram, it can be seen that very often an individual approach to the pathology of the anterior segment of the eye is necessary because many of these changes are unique to the individual patient, requiring planning to take into account all the presented features. We can evaluate these changes and take into account their potential impact on the procedure's result, thus obtaining the optimal effect with the lowest possible postoperative risk.

It should be remembered that changes in children can cause severe amblyopia; therefore, the basis of treatment planning is ensuring that the progression of amblyopia is as low as possible. Of course, we recognise that with advanced and severe diseases we are not always able to obtain the optimal effect and that we must sometimes employ indirect methods that are not wholly satisfactory but provide a minimum quality of vision. The consequences of a procedure and its potential complications are also worth considering. These can be quite numerous, so when planning any procedure, it should be decided whether the potential intervention may cause more complex disorders than leaving the eye's original state. If disorders are multifactorial and involve many tissues, interventions should not be undertaken rashly. It is worth checking and evaluating the evolution of changes over time, allowing for several examinations in finding an optimal time for intervention.

## 4. Discussion

The application of OCT in paediatric diagnostics dates back to the first time-domain OCT devices. Polish researchers were among the first to practically apply these new methods in diagnosing injuries. The proven usability of optical tomography has resulted in its widespread use. OCT Visante by Carl Zeiss Company was a breakthrough in imaging the anterior segment of the eye, in general, but it was not a portable device. The introduction of smaller devices, such as I-Vue by Optovue, and the embedding of optical tomography into ophthalmic microscopes have led to the common use of this imaging method in very young children and infants [4, 5].

Optical tomography is a tool for diagnosing the changes affecting each corneal layer. In the case of the epithelium, basement membrane defects and epithelial hyperplasia can be identified and located. These abnormalities are often accompanied by scars and fibrosis in the underlying corneal stroma, which are identified as hyperreflective areas often accompanied by corneal stromal thinning or an irregular anterior curvature. These disorders are dynamic, and epithelial recovery can improve the quality of vision, which can be confirmed in control examinations.

In addition to traumatic or inflammatory causes, neurotrophic keratopathy should always be considered when the dominant symptom is corneal epithelial defects. This disease is often responsible for epithelial regeneration disorders and the formation of shallow scars. It can be congenital or a result of corneal viral infections.

Cauduro et al. point out that in the case of paediatric diagnostics of acquired and congenital defects in the anterior segment, OCT provides invaluable services [6]. Optical tomography supports a topographic diagnosis of the anatomy of the anterior chamber that accounts for the position of the iris-lens diaphragm. A very important aspect, apart from the assessment of morphological changes in the cornea and morphometric changes of the anterior chamber, is the assessment of the filtration angle. It can be an important diagnostic guideline not only in assessing the risk of glaucoma but also during a therapeutic procedure.

Reduced corneal transparency and even its total leucoma are usually the features of congenital developmental disorders. Microcornea, sclerocornea, congenital corneal vasculature, relative anterior microphthalmia, microphthalmia, and Peters anomaly are the diseases which most often affect the cornea. These are often accompanied by changes in tissue thickness, causing an irregular anterior and posterior curvature of the cornea.

One aspect of the assessment of the eye globe is the analysis of the anterior chamber's depth and anatomy; Nakakura et al. analysed the accuracy of OCT, optical interferometry, Scheimpflug camera, and UBM in measuring the anterior chamber [7]. Their study showed the measurement discrepancies between devices. OCT was indicated as a very accurate method of imaging the anterior segment; the quality of its scans is no way inferior to that of the Scheimpflug camera and UBM. The authors point out that it is a noninvasive examination that can determine not only the depth but also the anatomy of the anterior chamber. This examination does not pose many limitations, and if it is possible to perform on a child, it is highly recommended.

Paediatric OCT examinations are necessary to prevent amblyopia, usually when funduscopic and gonioscopic evaluations are not eligible due to opaque optical media. OCT supports UBM, and together, they deliver high-quality scans which together support visualization of all anterior segment structures. Evaluations of congenital corneal and anterior chamber dysmorphia help to prognose a child's potential vision. If finding factors confirming potency for satisfactory visual acuity, a surgeon can consider a surgical approach for saving vision in infants or older children in, for example, traumatic cases [8].

One application of intraoperative OCT is keratoplasty in opaque corneas. Sharma et al. described surgery in children under 16 years of age on whom both diagnostics and treatment support were performed with an operating microscope with an integrated OCT device [9]. As the authors report, the device provided many benefits: detection of synechiae in the anterior chamber and precise determination of the anterior chamber's depth. This is a great advantage because it allows precise planning of the anterior chamber opening and further surgical management aimed at restoring the proper depth of the anterior chamber. The authors emphasize the diagnostic benefits, which support the course of surgery and finally optimize the postoperative outcome. Their findings were confirmed by Petrowicz et al., who used the OCT in an operating microscope to remove adhesions after a previous keratoplasty in the anterior chamber resulting in incomplete corneal transparency [10]. By using the OCT device, the procedure was less invasive and allowed for removal of the anterior chamber pathology without radical intervention. Eguchi et al. applied an integrated OCT to reduce the rate of anterior synechiae directly after keratoplasty was completed. Inspection at the end of the surgery allows the prevention of further interventions [11].

Our observations show that the use of OCT in the evaluation of congenital and acquired structural disorders of the eyeball is at the forefront among diagnostic devices. The noninvasive nature of the examination makes it possible to be performed during outpatient visits and under general anaesthesia. A wide range of devices with high diagnostic potential makes it a device that will become the basis of diagnostics, planning, and intraoperative treatment support techniques in the near future [12].

## Figures and Tables

**Table 1:** The main anterior segment disorders assessed during the study.

Anomaly	No. of cases	Main disorders	Main findings
Congenital loss of corneal transparency	23	Congenital leucomas, congenital glaucoma, corneal dystrophies, congenital corneal vasculature, sclerocornea dermoids in Goldenhar syndrome, isolated dermoids	Position and depth of hyperreflective corneal areas, corneal thinning or thickening, irregular corneal curvature, depth of scars or stromal fibrosis, structure of epithelium, stroma, and Descemet membrane
Acquired loss of corneal transparency	21	Leucomas and scars with infectious origin, immune disorders, posttraumatic lesions, complications of neurotrophic keratopathy	Position and depth of corneal pathology, involvement of particular corneal layers, irregularities of corneal shape
Iridocorneal disorders	17	Peters anomaly, Chandler's syndrome	Area of corneal involvement, stromal opacities, anterior and posterior curvatures, location of synechiae
Corneal shape irregularities	2	Ectasia, microcornea, megalocornea, microphthalmus	Irregularities of the anterior/posterior curvature and corneal thickness analysis
Eyes with surgical treatment history	9	Keratoplasty, PTK, keratectomy, congenital glaucoma surgery	Corneal transparency and thickness, anatomy of the anterior segment, implant position
Iridolenticular disorders	2	Persistent pupillary membrane, Axenfeld-Rieger anomaly, postinflammatory iridolenticular disorders	Anatomy of the anterior chamber, position of the iris, lens, synechiae

## Data Availability

The patients' data used to support the findings of this study are included within the article.
